# Treatment of Kawasaki Disease: A Network Meta-Analysis of Four Dosage Regimens of Aspirin Combined With Recommended Intravenous Immunoglobulin

**DOI:** 10.3389/fphar.2021.725126

**Published:** 2021-08-12

**Authors:** Ying-Hua Huang, Yi-Chen Hsin, Liang-Jen Wang, Wei-Ling Feng, Mindy Ming-Huey Guo, Ling-Sai Chang, Yu-Kang Tu, Ho-Chang Kuo

**Affiliations:** ^1^ Department of Pediatrics and Kawasaki Disease Center, Kaohsiung Chang Gung Memorial Hospital, Chang Gung University College of Medicine, Kaohsiung, Taiwan; ^2^ Department of Pediatric Allergy, Immunology, and Rheumatology, Division of Pediatrics, Chang Gung Memorial Hospital, Taoyuan, Taiwan; ^3^ Department of Child and Adolescent Psychiatry, Kaohsiung Chang Gung Memorial Hospital and Chang Gung University College of Medicine, Kaohsiung, Taiwan; ^4^ Institute of Epidemiology and Preventive Medicine, College of Public Health, National Taiwan University, Taipei, Taiwan

**Keywords:** acetylsalicylic acid, aspirin, Kawasaki disease or Kawasaki syndrome, mucocutaneous lymph node syndrome, salicylate

## Abstract

Aspirin was once believed to reduce the mortality of Kawasaki disease (KD) due to its effect on the thrombotic occlusion of coronary arteries. However, conflicting evidence has been found regarding aspirin treatment and its benefit in patients with acute KD. We compared the efficacy of different aspirin doses in acute KD. A literature search of PubMed, EMBASE, and Cochrane databases was conducted to identify studies comparing different doses of aspirin for acute KD. The primary outcome of interest was coronary artery lesions (CAL). We used random-effects network meta-analysis. Six retrospective studies, including 1944 patients receiving aspirin in doses of 0, 3–5, 30–50, or 80–100 mg/kg/day, were selected. The risks of CAL were not significantly different for the various doses of aspirin compared to the placebo: odds ratio (OR) was 1.10 [95% confidence interval (CI): 0.70–1.71] for patients with aspirin 3–5 mg/kg/day; OR = 1.23 (95% CI: 0.67–2.26) for aspirin 30–50 mg/kg/day, and OR = 1.59 (95% CI: 0.74, 3.421) for 80–100 mg/kg/day. The P-score ranged from 0.76 for placebo to 0.19 for aspirin 80–100 mg/kg/day. The different doses of aspirin exhibited no significant difference with regard to the efficacy of CAL or with the secondary outcomes of intravenous immunoglobulin resistance or hospital stays for acute KD. Therefore, we found that treatment without any aspirin is not inferior to other doses of aspirin and can also slightly reduce the risk of CAL.

## Introduction

Pediatrician Kawasaki Tomisaku first published a report in 1967 on about 50 patients who presented with persistent fever, rash, lymphadenopathy, edema of the limbs, conjunctival injection, redness, and cracking of the lips, strawberry tongue, and convalescent desquamation (Kawasaki, 1967). Since then, acute febrile infantile mucocutaneous lymph node syndrome has also been referred to as Kawasaki disease (KD) because this distinct clinical entity had never been clearly defined before (Tanaka, 1975). The correlation between KD and coronary vasculitis was later established, with KD being classified as a type of medium vasculitis in a symposium held on systemic vasculitis held at Chapel Hill, North Carolina in 1994 (Burns, 2018). Histopathological investigation of the vascular findings in autopsies of KD patients with sudden cardiac death also demonstrated coronary arteritis (Roberts and Fetterman, 1963; Kushner and Abramowsky, 2010).

The treatment guidelines published by the American Heart Association in 2017 recommend that the standard treatment for acute-phase KD was high-dose intravenous immunoglobulin (IVIG) (level of evidence A) plus acetylsalicylic acid (aspirin) (level of evidence C) (McCrindle et al., 2017). However, the reason for recommending aspirin use is actually because the experimental group and the control group often use the same dose of aspirin in clinical trials, which has prevented clinicians from truly clarifying the aspirin that KD patients genuinely need. Appropriate treatment can reduce coronary artery aneurysm incidence on the 30th day. A single dose of IVIG >1 g/kg is more effective than multiple doses of IVIG for preventing 30 days of coronary artery aneurysm (Durongpisitkul et al., 1995). IVIG >1 g/kg plus ≤ 80 mg/kg/day aspirin is as effective as IVIG >1 g/kg plus aspirin >80 mg/kg/day in preventing coronary artery aneurysm (Durongpisitkul et al., 1995). The effect of preventing coronary artery aneurysm is proportional to the total dose of IVIG, which means that the total dose of 2 g/kg of IVIG is best (Terai and Shulman, 1997). Under the same IVIG dose, no statistical difference has been found in the effectiveness of aspirin at 30–50 mg/kg/day and aspirin at 80–120 mg/kg/day in preventing coronary artery abnormalities (Terai and Shulman, 1997). Due to the continued recommendation of aspirin, analysis in the relevant literature regarding aspirin not being used in the acute phase of KD is lacking.

In contrast, despite a lack of evidence provided by randomized control trials (RCT), traditional high-dose aspirin was considered to have no significant therapeutic effect in a 10-years retrospective study of 260 KD children (Platt et al., 2020). However, understanding the preventive effect of low-dose aspirin on coronary artery lesions (CAL) is still necessary (Chiang et al., 2020). Kuo et al. are currently conducting a comparative study on the effectiveness of IVIG alone and high-dose aspirin (80–100 mg/kg/day) as treatment in the acute phase of KD. The study has been designed as a multi-center, prospective, randomized controlled double-blind trial with two parallel groups to determine whether IVIG alone as the main treatment for acute KD is as effective as the combined treatment with high-dose aspirin. The endpoint of said trial is the formation of CAL observed at 6–8 weeks (Kuo et al., 2018).

According to previous studies by Kuo et al., using high-dose aspirin (80–100 mg/kg/day) in the acute phase of KD has demonstrated no significant benefit, and the incidence of CAL and the number of days of hospitalization between high and low-dose aspirin also showed no significant difference. However, high-dose aspirin has affected the recovery of hemoglobin. The results of this study revealed the controversy over high-dose aspirin use in the acute phase of KD (Kuo et al., 2015). In a retrospective study with a large sample size, the use of aspirin was most prone to side effects related to the digestive system (5.3% among 910 KD patients) (Huang et al., 2018). Bleeding in the upper and lower gastrointestinal tract and abnormal liver function have been reported, and symptoms of abdominal discomfort may also appear in patients treated with aspirin (Matsubara et al., 1996; Zheng et al., 2019; Mammadov et al., 2020). Asthma caused by aspirin was also a suspected reason for the increase in the risk of asthma following KD (Kawane et al., 1996; Van Bever et al., 2004; Huang et al., 2020).

In the plan of a RCT, due to sample size considerations, designing multiple arms with four commonly used doses (placebo, 3–5, 30–50, 80–100 mg/kg/day) was impractical and not feasible. Furthermore, several meta-analysis comparisons consisted primarily of low- and high-dose comparisons (Zheng et al., 2019; Chiang et al., 2020; Jia et al., 2020). We still do not know whether aspirin is necessary for treating KD in the acute phase or the best dose of treatment, so the diagnosis and treatment guidelines are currently incapable of providing appropriate recommendations based on evidence (McCrindle et al., 2017). Therefore, it is necessary to use network meta-analysis to compare the effects of various commonly used treatment doses of aspirin administered in conjunction with 2 g/kg IVIG.

## Materials and Methods

This network meta-analysis was reported in accordance with the general principles of the Preferred Reporting Items for Systematic Reviews and Meta-Analysis (PRISMA) extension to network meta-analysis (Hutton et al., 2015). We adopted a population–intervention–comparison–outcome framework for study inclusion in order to describe the treatment of acute Kawasaki disease, the impact of aspirin by dose plus 2 g/kg IVIG in the therapeutic outcomes of CAL, IVIG resistance, and hospital stays.

### Search Strategy

For our comprehensive search, we systematically scanned the following databases from inception to April 30, 2021: Cochrane Database of Systematic Reviews (*n* = 3), Embase (*n* = 3,573), and Pubmed (*n* = 973). Our search strategy was developed in Pubmed with input from the review team. The Medical Subject Headings (MeSH) used in the search strategy included [Mucocutaneous Lymph Node Syndrome (MeSH Terms) OR Kawasaki disease OR Kawasaki syndrome] AND [aspirin (MeSH Terms) OR acetylsalicylic acid OR salicylate]. Searches were not limited by language or study design. The Pubmed strategy was also applied to all the other resources searched. Search results were imported into EndNote X9 (Clarivate Analytics, Philadelphia, PA, United States) and de-duplicated.

We carried out a search for studies of different aspirin doses and updated searches as necessary. In view of limitations on time and resources, we decided to identify studies by specifically searching relevant systematic reviews and meta-analyses.

Studies were initially assessed for relevance using titles and abstracts. When possible, we obtained full manuscripts of any titles/abstracts that appeared to be relevant, and two reviewers independently assessed the relevance of each study.

Relevant studies were evaluated for quality, and key outcome data were extracted. The outcome measures to be considered included the incidence of CAL as the primary outcome, with IVIG resistance and hospital stays as secondary outcomes. The incidence of CAL was assessed through echocardiograph. All coronary artery abnormalities, including either small aneurysm or dilatation, were considered CAL. If the same study had two CAL definition methods, we recorded the stricter and more consolidated one. Using a standardized data form in Microsoft Excel, two reviewers (LSC and YHH) independently extracted and tabulated data for study quality, intervention characteristics, participants, and outcomes from the final list of selected eligible studies. If any discrepancies were observed between the studies selected by the same investigators, a third investigator (YCH) was involved through consensus.

### Selection Criteria and Data Analysis

When sufficient data were available for quantitative assessment, we applied a network meta-analysis (NMA). The population of interest focused on human studies in which all subjects were diagnosed with KD. For all doses of aspirin in the acute phase of KD, we performed a NMA for relevant comparators among aspirin 0, 3–5, 30–50, and 80–100 mg/kg/day. Evidence on combined treatments (e.g., 2 g/kg IVIG plus indicated aspirin doses) was considered for inclusion. The acute phase of KD was defined as the period that started at the febrile phase after KD was diagnosed and ended with defervescence according to the American Heart Association (McCrindle et al., 2017). We did not consider other studies using any doses of aspirin or IVIG that were not indicated as part of the NMA.

We conducted random effects NMA by using R software’s “netmeta” package. The estimates of effectiveness relative to the reference treatment were summarized using odds ratio with accompanying 95% confidence intervals (CI) for CAL and IVIG resistance. The estimates of effectiveness were measured using mean differences with accompanying 95% CI for our NMA on days of hospital stays. The treatments were ranked using P‐score. Where possible, consistency between direct and indirect estimates of treatment effect in the NMA was assessed using the design-by-treatment interaction model and node-splitting model (Yu-Kang, 2016). The quality of the nonunionized studies included in the meta-analyses was determined using the Newcastle-Ottawa Scale (NOS) (Baumer et al., 2006). Using said scale, studies that scored at least five stars were considered to have moderate to high methodological quality. Quality assessment was undertaken by one investigator and then independently checked by a second investigator.

I^2^ values can range from 0 to 100%; a value greater than 75% represents high heterogeneity, while one less than 25% suggests low heterogeneity (Jia et al., 2020).

## Results

**FIGURE 1:**
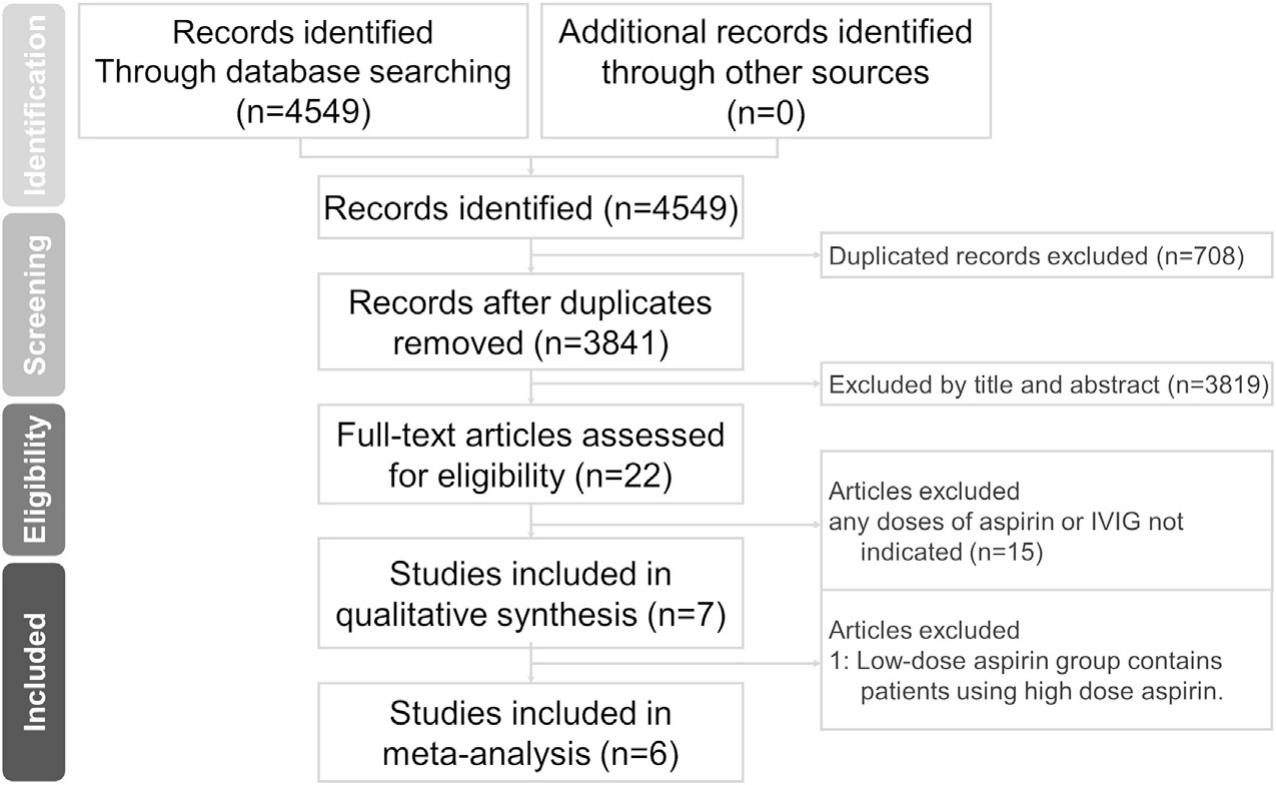
Preferred Reporting Items for Systematic Reviews and Meta-Analyses (PRISMA) flow diagram of the study search and selection process. IVIG, intravenous immunoglobulin.

The electronic database searches and manual search of reference lists from published reviews for clinical effectiveness evidence on different aspirin doses (i.e., 0, 3–5, 30–50, 80–100 mg/kg/day) yielded a total of 3,841 abstracts published between 1978 and 2021 after de-duplication between databases (Figure 1). We had obtained full papers and checked reference lists from records that were potentially relevant systematic reviews or meta-analyses (Durongpisitkul et al., 1995; Terai and Shulman, 1997; Baumer et al., 2006; Ho and Curtis, 2017; Zheng et al., 2019; Chiang et al., 2020; Jia et al., 2020). A total of 3,819 studies were ruled out after screening the titles and abstracts.

Of these, 22 studies underwent a further full-text review for eligibility, and six retrospective comparative studies ultimately met our inclusion criteria. The study by Dallaire was eliminated from the analysis since it included 24/365 subjects with aspirin dose >10 mg/kg/day in the low-dose aspirin group after excluding patients without IVIG (Dallaire et al., 2017). A small number of patients also received steroid or infliximab treatment. Ito et al. compared aspirin 30 mg/kg/day along with 2 g/kg IVIG to 50 mg/kg/day combined the same IVIG dose (Ito et al., 2020). Kim et al. compared two groups between KD patients with aspirin 3–5 and ≥30 mg/kg/day (Kim et al., 2017). All subjects were prescribed 2 g/kg IVIG. Kuo et al. defined high-dose aspirin >30 mg/kg/day and compared it with no aspirin use (Kuo et al., 2015). The patients were treated with a single dose of IVIG (2 g/kg). Another dual-center retrospective study by Kuo et al. compared the therapeutic effects of high-dose aspirin >50 mg/kg/day and low-dose 3–5 mg/kg/day (Kuo et al., 2012). All patients in both groups were treated with the standard care of IVIG. In the study by Platt et al., 10 mg/kg/day was regarded as the cut-off value for high and low doses of aspirin (Platt et al., 2020). Most patients received the Privigen brand of IVIG. Studies by Akagi, Koren, Saulsbury, Wang, and others all had KD patients treated with IVIG at doses other than 2 g/kg (Koren et al., 1985a; Akagi et al., 1990; Akagi et al., 1991; Saulsbury, 2002; Wang et al., 2020). Akagi et al. investigated the therapeutic efficacy of 100 vs. 30 mg/kg/day aspirin (Akagi et al., 1990). Koren et al. compared whether KD patients with and without aspirin 80–180 mg/kg/day developed coronary artery aneurysms (Koren et al., 1985a). In the study by Saulsbury et al., patients receiving IVIG were divided into 400 mg/kg for four consecutive days or a single infusion of 2 g/kg, and aspirin treatment was divided into 80–100 or 3–5 mg/kg/day (Saulsbury, 2002). The retrospective study of Wang et al. divided aspirin into the following three groups: 20–29, 30–39, and 40–50 mg/kg/day (Wang et al., 2020). Migally et al. did not clearly define what dose patients without high-dose aspirin received, while patients who did not receive IVIG were also included in the study (Migally et al., 2018). Studies performed before the beginning or suggestion of IVIG treatment were excluded (Yokoyama et al., 1980; Koren et al., 1985b; Ichida et al., 1987). This study did not exclude any papers from its analysis for the reason that the full-text data was not available.

**FIGURE 2:**
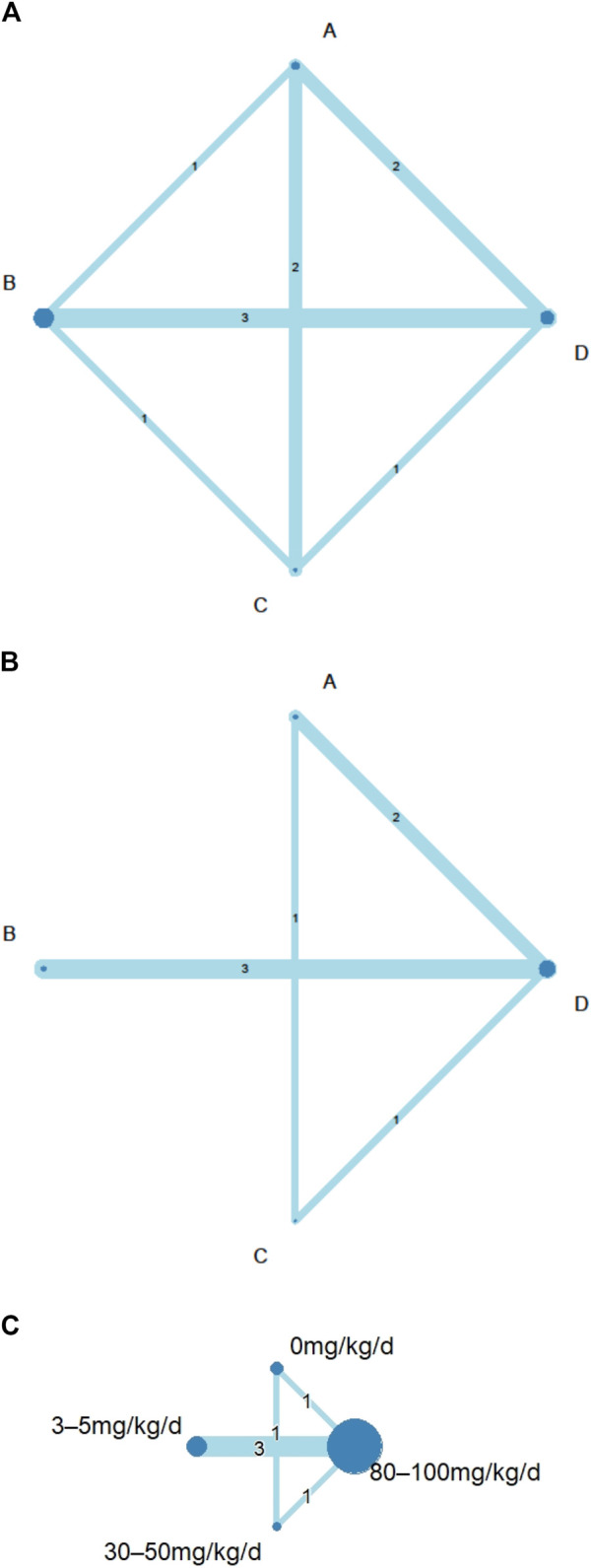
Network geometry of eligible comparisons among different doses of aspirin for: **(A)** consistency analyses of coronary artery lesions (CAL) rate by node-split model; **(B)** intravenous immunoglobulin (IVIG) resistance rate; **(C)** length of hospital stays. Network diagrams were produced with node size corresponding to the number of included studies and the line width representing the number of included studies comparing the interventions. The number of direct comparisons was expressed as a number in the middle of a line between nodes. **(A)** aspirin 0 mg/kg/day; **(B)**, 3–5 mg/kg/day; **(C)**, 30–50 mg/kg/day; **(D)**, 80–100 mg/kg/day; d, day.

In total, the included studies consisted of 1944 participants that we divided into four unique doses (Figure 2). The list of the final subset is summarized in Table 1. The studies were conducted primarily in Asia, with the following breakdown: Korea (*n* = 2), Japan (*n* = 1), China (*n* = 1), Iran (*n* = 1), and Canada (*n* = 1) (Lee et al., 2013; Rahbarimanesh et al., 2014; Amarilyo et al., 2017; Dhanrajani et al., 2018; Huang et al., 2018; Kwon et al., 2020). Two of the studies investigated three different doses, whereas the other studies investigated only two different doses.

**TABLE 1:** Study characteristics and information of the six included studies.

References first author year published	Country and number of centers	Study characteristic	Endpoints	Total number of patients	Aspirin mg/kg/day	Age (months)	Male (%)	NOS
Kwon et al., 2020 (Kwon et al., 2020)	Korea One	retrospective cohort	CAL	323	0	39.6	55.6	7
			IVIG R		30–50	34.2	59.3	
			Hospital stays		80–100	32.8	68	
Huang et al., 2018 (Huang et al., 2018)	China One	retrospective cohort	CAL	910	0	22.8	67	8
					3–5	23.7	63	
					30–50	25.8	69	
Dhanrajani et al., 2018 (Dhanrajani et al., 2018)	Canada Two	retrospective chart review	CAL	242	3–5	33	59	7
			IVIG R					
			Hospital stays		80–100	36	58.3	
Amarilyo et al., 2017 (Amarilyo et al., 2017)	Israel	retrospective, cohort study	CAL	220	3–5	34.8	65.1	6
			IVIG R					
	Six		Hospital stays		80–100	28.8	63.5	
Rahbarimanesh et al., 2014 (Rahbarimanesh et al., 2014)	Iran One	Not reported	CAL	69	3–5	—	—	4
			IVIG R		80–100			
			Hospital stays					
Lee et al., 2013 (Lee et al., 2013)	Korea One	Not reported	CAL	180	0	30.7	58.82	6
			IVIG R		80–100	30.2	55.81	

CAL, coronary artery lesions; IVIG R, resistance to intravenous immunoglobulin; NOS, Newcastle-Ottawa Scale.

### Coronary Artery Lesions

**FIGURE 3:**
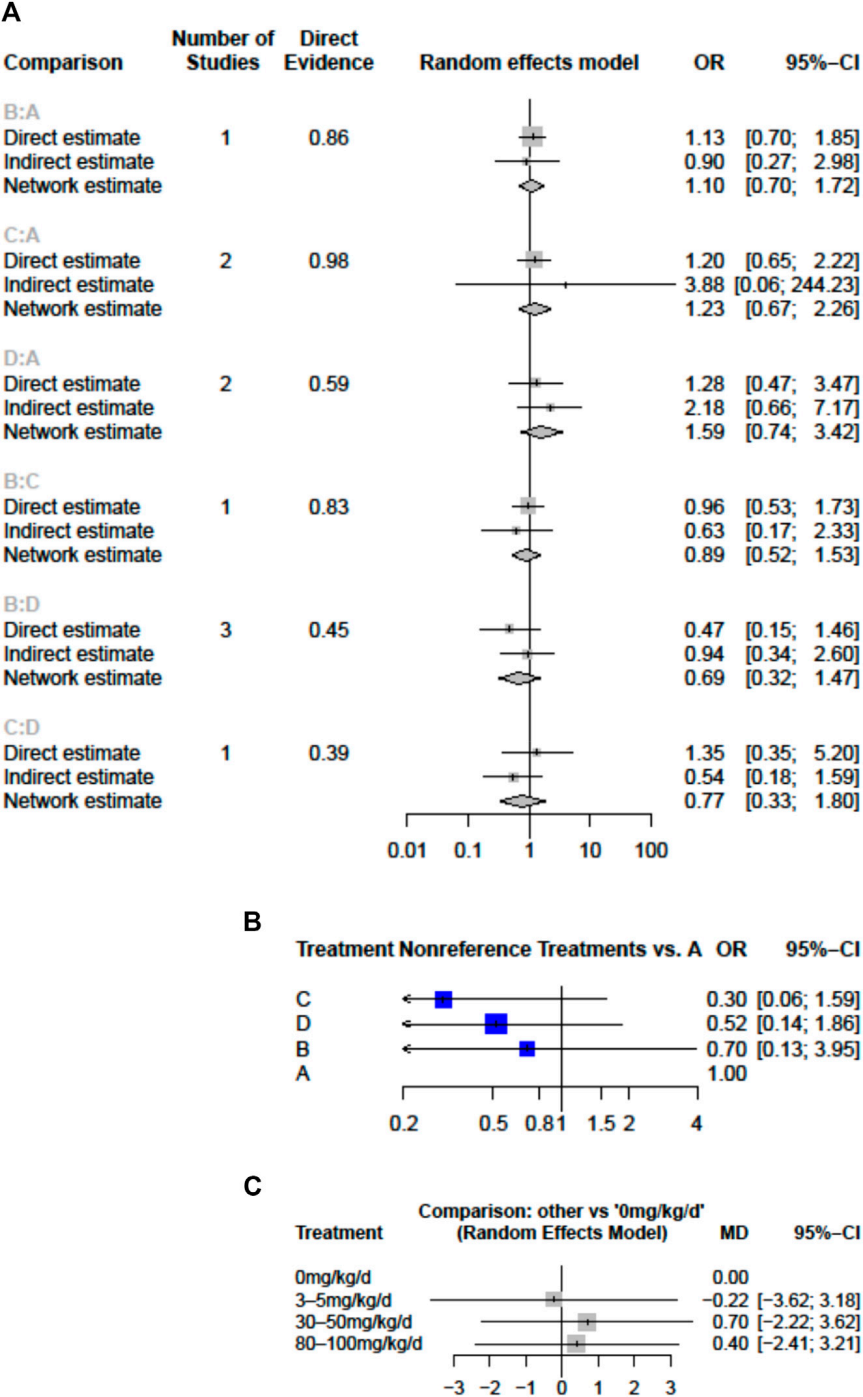
Forest plots of network meta-analysis for the effects of aspirin doses on **(A)** the risk of coronary artery lesions (CAL) **(B)** the risk of intravenous immunoglobulin (IVIG) resistance **(C)** hospital stays (mean difference in days) **(A)**, aspirin 0 mg/kg/day **(B)**, 3–5 mg/kg/day **(C)**, 30–50 mg/kg/day; D, 80–100 mg/kg/day; CI, confidence interval; d, day; OR, odds ratio.

All doses of aspirin had a similar effect on CAL outcome. We did not detect significant inconsistencies in the evidence networks for any of the secondary outcomes. Applying a random-effects model, the results for all the individual treatment comparisons (both direct and indirect), are shown in Figure 3. No significant difference was noted between 30–50 mg/kg/day (compared to placebo, OR = 1.23, 95% CI: 0.67–2.26) and between 80–100 mg/kg/day and placebo (OR = 1.59, 95% CI: 0.74–3.42). An I^2^ statistic of 0% indicated that heterogeneity was low for the included studies. No inconsistency was observed between direct and indirect comparisons in the loop-specific analysis of CAL (*p* = 0.8072). Furthermore, one of the retrospective comparative studies was rated as four stars and was considered to be of low quality, while the other five reports were awarded ≧ six stars and qualified as having high quality.

### IVIG Resistance

**FIGURE 4:**
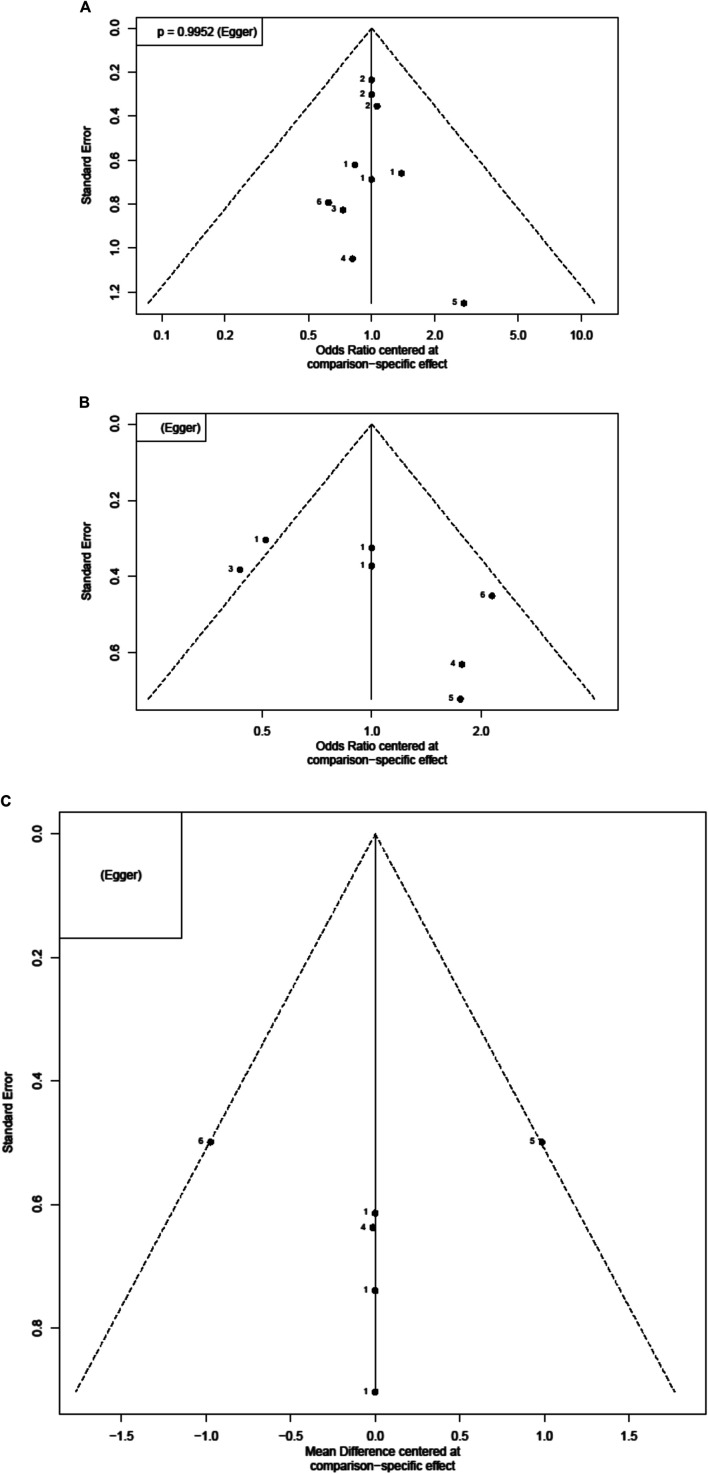
Funnel plots for each outcome. Each dot reflects a study; the *y*-axis reflects the sample size or standard error, and the *x*-axis reflects the effect size of each study. Large studies are distributed in the top of the plot, and smaller studies are scattered toward the bottom of the plot **(A)** risk of coronary artery lesions (CAL) **(B)** risk of intravenous immunoglobulin (IVIG) resistance **(C)** length of hospital stays.

IVIG-resistant KD patients are defined as patients that need to be treated again. Possible options are a second dose of IVIG, infliximab, or intravenous pulse methylprednisolone (Chan et al., 2019). Re-treatment prolongs the length of hospital stay and costs and is also a risk factor for CAL (Chang et al., 2020). The evidence basis of our NMA consisted of five studies involving 1,179 children (Figure 2). Compared to placebo, any aspirin given to KD patients, such as 3–5, 30–50, and 80–100 mg/kg/day, showed a decreasing trend in the prevalence of IVIG resistance, but its CI overlapped with the null effect of one. Regarding IVIG resistance, no meaningful risk difference was identified among these four groups. Aspirin 3–5, 30–50, and 80–100 mg/kg/day were found to be comparable to placebo (Figure 3). We conducted pairwise meta-analysis for the different aspirin regimens based on dosing (Supplementary Table S1). Among the interventions, aspirin 30–50 mg/kg/day ranked highest for the prevention of IVIG resistance, followed by 80–100, 3–5 mg/kg/day, and the placebo. An I^2^ statistic of 75.5% indicated substantial heterogeneity in this model. Visual inspection for publication bias, which was detected using funnel plots, demonstrated asymmetry in Figure 4.

### Length of Hospitalization

Four studies with a total of 999 participants reported the duration of hospital stay (Figure 2). The mean duration of hospital stays ranged from 7.3 days in the aspirin 80–100 group compared to the 3–5 mg/kg/day group in the study by Amarilyo et al. to 4.1 days in the 3–5 mg/kg/day group compared to aspirin 80–100 in the study by Dhanrajani et al. (Supplementary Table S2). KD patients using aspirin 3–5 mg/kg/day showed the shortest hospital stays. Compared to placebo, the combination of aspirin 3–5 mg/kg/day and IVIG showed a mean difference (MD) of −0.22 days (95% CI: −3.62, 3.18) for hospital stays. Upon analyzing the individual studies, the MD in length of hospitalization was not statistically significant for any of the direct comparisons; by evaluating the MD values for each comparison, all confidence intervals crossed the zero value (Figure 3 and Supplementary Table S3). Evaluating the P-score, the values ranged from 0.6853 for aspirin 30–50 mg/kg/day to 0.2840 for aspirin 3–5 mg/kg/day (Supplementary Table S4). After applying a random-effects model, the results for all the individual treatment comparisons (both direct and indirect), are shown in Figure 3, Supplementary Table S3, and Supplementary Figure S1. Visual inspection of the symmetrical funnel plot of hospital stays indicated no publication bias (Figure 4).

In comparing moderate doses (30–50 mg/kg/day) and high doses (80–100), the length of hospital stay at a moderate dose was slightly higher by MD 0.30 (−2.22–2.82) (Supplementary Table S3). However, the risk of CAL (OR, 0.77; 95% CI 0.33–1.80) and the chance of needing to be treated again due to IVIG resistance (Supplementary Table S1) were both slightly lower in KD patients who received moderate-dose aspirin treatment. The comparison of 0 and 3–5 mg/kg/day (low dose) and 30–50 and 80–100 mg/kg/day (high dose) of aspirin found that the risks of CAL and IVIG resistance did not differ significantly.

In summary, from P-scores and forest plots, 3–5 mg/kg/day aspirin can numerically reduce the length of hospital stays. However, the data did not reach statistical differences. The mean difference of the length of hospital stay was small, i.e., less than 1 day.

## Discussion

To the best of our knowledge, this study is the first to identify effects of different aspirin doses with regard to CAL, IVIG resistance, and hospital stays in KD using NMA. Although aspirin has been widely used in the treatment of KD, no optimal dose has yet been confirmed. Studies have found that KD children with aspirin 80–100 mg/kg/day had a lower risk of developing refractory KD and tend to have shorter fever duration (Lee et al., 2013; Dhanrajani et al., 2018). Ito and his colleague investigated KD outcomes for aspirin 30 and 50 mg/kg/day, respectively. The risk of refractory KD in the 30 mg/kg/day aspirin group was significantly higher than the 50 mg/kg/day aspirin (odds ratio 1.379, *p* = 0.021). Even when the difference in aspirin is only 20 mg/kg/day, different therapeutic effects may be obtained. Because many studies have not strictly defined the dose of aspirin, a robust comparison of these different doses remains incomplete.

In previous studies, results showing aspirin efficacy for KD have varied. The use of moderate-dose aspirin has been recommended because no aspirin may be associated with a higher chance of receiving a second treatment, and 80–100 mg/kg/day aspirin may have life-threatening side effects (One patient complicated with Reye’s syndrome died) (Kwon et al., 2020). Moderate or high-dose aspirin was unable to reduce the CAL risk in KD children compared to 3–5 mg/kg/day aspirin in the retrospective Korean nationwide survey (Kim et al., 2017). In accordance with our analysis, it has been suggested that aspirin <10 mg/kg/day combined with IVIG may be as effective as aspirin ≥10 or >30 mg/kg/day plus IVIG (Jia et al., 2020; Platt et al., 2020). In addition, prescribing low-dose 3–5 mg/kg/day or no aspirin for the initial treatment of KD may be associated with a decreased incidence of CAL compared to aspirin ≥30 mg/kg/day (Zheng et al., 2019; Chiang et al., 2020; Jia et al., 2020).

The mechanism of action of low-dose aspirin is anti-platelet aggregation, which employs the irreversible inhibition of platelet cyclooxygenase to prevent arachidonic acid from converting into thromboxane A2, thus reducing platelet aggregation and release response. Regarding KD treatment, high-dose aspirin was administered from 80 to 100 mg/kg/day in the US to 30–50 mg/kg/day in Japan and then changed to low-dose aspirin, 3–5 mg/kg/day, 48 h after defervescence for 6–8 weeks (Chang et al., 2017). The mechanisms of action of high-dose aspirin greater than 30 mg/kg/day are anti-inflammatory, analgesic, and antipyretic (Kato et al., 1979). Aspirin inhibits cyclooxygenase in a similar manner as other NSAIDs, while high-dose aspirin in particular inhibits the cyclooxygenase vinylation of the arterial wall and interferes with the production of powerful vasodilators and platelet aggregation inhibitor prostacyclin. Prostaglandin and thromboxane B2 (TxB2) were significantly decreased after treatment with an aspirin dose of 30 mg/kg/day (Sasai, 1988; Tanoshima et al., 2019). The TxB2/6-keto- prostaglandin F1 (PGF1) ratio was decreased with the aspirin dose of 2–5 mg/kg (*p* < 0.05) (Tanoshima et al., 2019). Akagi et al. found that high-dose aspirin therapy (100 mg/kg/day) may be disadvantageous as anti-thrombotic treatment because the surveyed plasma TxB2 production, which was completely blocked in patients with aspirin doses of 30 or 100 mg/kg/day, and plasma 6-keto-PGF1 alpha levels in patients with 100 mg/kg/day on day 14 were lower than those in patients with an aspirin does of 30 mg/kg/day (Akagi et al., 1990). An investigation of eicosanoid metabolism identified significantly lower TxB2 in KD patients with aspirin doses of 30 or 60 mg/kg/kg compared with no aspirin (Fulton et al., 1988). Salicylate bioavailability correlated to serum concentrations was impaired during the acute phase and then significantly increased during the subacute phase. Remarkably lower serum albumin concentrations resulted in decreased protein binding in the acute phase and more free salicylates (Koren et al., 1988; Koren et al., 1991). Aspirin doses lower than 80 mg/kg/day could not reach the therapeutic concentration; meanwhile, more than a quarter of patients with aspirin >120 mg/kg/day exceeded toxic concentration (Koren and MacLeod, 1984). Another route of aspirin dose above 100 mg/kg/day with intravenous administration posed a beneficial anti-inflammatory effect (Umezawa et al., 1992). However, aspirin may cause stomach bleeding, especially when combined with alcoholic beverages. Combined with chickenpox or influenza, aspirin can cause Reye’s syndrome, which has a mortality rate of up to 30% (Matsumoto et al., 2020). Furthermore, in patients with favism, aspirin may cause hemolytic anemia (Chen et al., 2014).

This NMA had several limitations. First, due to the different definitions of fever reduction (calculated from the beginning of IVIG treatment or the end of treatment), the comparison of the NMA was not possible (Lee et al., 2013; Rahbarimanesh et al., 2014; Kwon et al., 2020). Second, due to the retrospective study design, estimating the adverse effects of aspirin was difficult, so this NMA could not compare the adverse effects of different doses of aspirin. Furthermore, pharmacogenomic differences among patients of different races could have been a confounding variable (Mallah et al., 2020).

The body of evidence on the clinical effectiveness and safety of different aspirin doses is small, especially with regard to a lack of RCT, but our NMA indicates similar effects among different aspirin doses in their influence of KD prognosis. Our review does not support using low-dose (3–5 mg/kg/day), moderate (30–50 mg/kg/day), or high-dose (80–100 mg/kg/day) aspirin in the acute phase of KD. Similar to the conclusion of Hsieh et al., no aspirin is not inferior to other doses of aspirin and can also slightly reduce the risk of CAL (Hsieh et al., 2004). Further prospective RCT studies are warranted to confirm the efficacy and adverse events of different doses of aspirin treatment for acute KD.

## Data Availability

The original contributions presented in the study are included in the article/Supplementary Material, further inquiries can be directed to the corresponding authors.
