# Supplementary material for: Treatment of Kawasaki Disease: A Network Meta-Analysis of Four Dosage Regimens of Aspirin Combined With Recommended Intravenous Immunoglobulin
Source: Front Pharmacol. 2021 Aug 12;12:725126. doi: 10.3389/fphar.2021.725126 (PMC8397445; doi:10.3389/fphar.2021.725126)
Supplement: Supplementary file 1 [file DataSheet1.docx]

1. **Supplementary Figures and Tables**

**1.1 Supplementary Tables**

**Supplementary Table 1.** Odds Ratios for the risk of intravenous immunoglobulin resistance.

|  | V1 | V2 | V3 | V4 |
| --- | --- | --- | --- | --- |
| 1 | 30-50 mg/kg/day | 0.82 (0.13 to 5.00) | . | 0.22 (0.04 to 1.27) |
| 2 | 0.58 (0.11 to 3.16) | 80-100 mg/kg/day | 0.73 (0.23 to 2.33) | 0.52 (0.14 to 1.86) |
| 3 | 0.42 (0.05 to 3.30) | 0.73 (0.23 to 2.33) | 3-5 mg/kg/day | . |
| 4 | 0.30 (0.06 to 1.59) | 0.52 (0.14 to 1.86) | 0.70 (0.13 to 3.95) | 0 mg/kg/day |

**Supplementary Table 2.** Estimations of mean hospital stays and standard deviation (sd) for each treatment regimen (txt). d, day.

study treat1 treat2 TE seTE n1 mean1 sd1 n2 mean2 sd2

1 1 30–50 mg/kg/d 0 mg/kg/d 0.70 0.7397950 91 6.20 6.70 135 5.5 2.70

2 1 30–50 mg/kg/d 80–100 mg/kg/d 0.30 0.9036568 91 6.20 6.70 97 5.9 5.60

3 1 80–100 mg/kg/d 0 mg/kg/d 0.40 0.6142467 97 5.90 5.60 135 5.5 2.70

4 4 3–5 mg/kg/d 80–100 mg/kg/d -0.60 0.6376552 122 4.10 5.03 127 4.7 5.03

5 5 3–5 mg/kg/d 80–100 mg/kg/d -1.60 0.4994999 43 5.70 2.80 315 7.3 4.60

6 6 3–5 mg/kg/d 80–100 mg/kg/d 0.36 0.4992587 42 6.36 2.80 27 6.0 1.30

id study1 study2 Aspirin1 Aspirin2 mean.orig1

1 1 Kwon 2020 Kwon 2020 30–50 mg/kg/d 0 mg/kg/d 6.20

2 1 Kwon 2020 Kwon 2020 30–50 mg/kg/d 80–100 mg/kg/d 6.20

3 1 Kwon 2020 Kwon 2020 80–100 mg/kg/d 0 mg/kg/d 5.90

4 4 Dhanrajani 2018 Dhanrajani 2018 3–5 mg/kg/d 80–100 mg/kg/d 4.10

5 5 Amarilyo 2017 Amarilyo 2017 3–5 mg/kg/d 80–100 mg/kg/d 5.70

6 6 Rahbarimanesh 2014 Rahbarimanesh 2014 3–5 mg/kg/d 80–100 mg/kg/d 6.36

mean.orig2 sd.orig1 sd.orig2 n.orig1 n.orig2 txt1 txt2

1 5.5 6.70 2.70 91 135 30–50 mg/kg/d 0 mg/kg/d

2 5.9 6.70 5.60 91 97 30–50 mg/kg/d 80–100 mg/kg/d

3 5.5 5.60 2.70 97 135 80–100 mg/kg/d 0 mg/kg/d

4 4.7 5.03 5.03 122 127 3–5 mg/kg/d 80–100 mg/kg/d

5 7.3 2.80 4.60 43 315 3–5 mg/kg/d 80–100 mg/kg/d

6 6.0 2.80 1.30 42 27 3–5 mg/kg/d 80–100 mg/kg/d

**Supplementary Table 3.** League Table of pair-wise comparisons in the network meta-analysis for the mean difference of hospital stays.

|  | V1 | V2 | V3 | V4 |
| --- | --- | --- | --- | --- |
| 1 | 30–50 mg/kg/day | 0.30 (-2.22 to 2.82) | 0.70 (-1.60 to 3.00) | . |
| 2 | 0.30 (-2.22 to 2.82) | 80–100 mg/kg/day | 0.40 (-1.76 to 2.56) | 0.61 (-0.59 to 1.82) |
| 3 | 0.70 (-1.60 to 3.00) | 0.40 (-1.76 to 2.56) | 0 mg/kg/day | . |
| 4 | 0.91 (-1.88 to 3.71) | 0.61 (-0.59 to 1.82) | 0.21 (-2.26 to 2.68) | 3–5 mg/kg/day |

**Supplementary Table 4** Relative ranking of the effect of different doses on the mean difference of hospital stays assessed by P-score.

P-score

30–50 mg/kg/day 0.6853

80–100 mg/kg/day 0.6302

0 mg/kg/day 0.4004

3–5 mg/kg/day 0.2840

**1.2 Supplementary Figures**


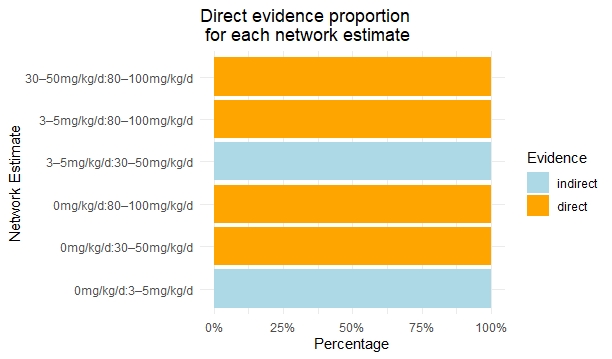


**Supplementary Figure 1.** Direct evidence proportion for each network estimate. d, day
